# The Key to Individualized Addiction Treatment is Comprehensive Assessment and Monitoring of Symptoms and Behavioral Change

**DOI:** 10.3390/bs5040477

**Published:** 2015-10-30

**Authors:** Thomas F. Hilton, Paul A. Pilkonis

**Affiliations:** 1 101 Martesia Way, Indian Harbour Beach, FL 32937, USA; 2 Western Psychiatric Institute & Clinic, University of Pittsburgh Medical Center, 3811 O’Hara Street Pittsburgh, PA 15213, USA; E-Mail: pilkonispa@upmc.edu

**Keywords:** addiction, recovery, monitoring, NIH PROMIS, NIH Toolbox, individualized medicine

## Abstract

Modern health services now strive for individualized treatment. This approach has been enabled by the increase in knowledge derived from neuroscience and genomics. Substance use disorders are no exception to individualized treatment even though there are no gene-specific medications yet available. What is available is the ability to quickly and precisely assess and monitor biopsychosocial variables known to vary during addiction recovery and which place addicts at increased risk of relapse. Monitoring a broad spectrum of biopsychosocial health enables providers to address diverse genome-specific changes that might trigger withdrawal from treatment or recovery relapse in time to prevent that from occurring. This paper describes modern measurement tools contained in the NIH Patient-Reported Outcomes Measurement Information System (PROMIS) and the NIH Toolbox and suggests how they might be applied to support recovery from alcohol and other substance use disorders in both pharmacological and abstinence-oriented modalities of care.

## 1. Introduction

For almost two decades, leaders in the substance use disorder (SUD) field have called for a shift from an acute care model of addiction treatment to a chronic care model in which recovery—not abstinence alone—is the goal [1,2]. Although treatment programs have been moving toward a chronic care model, resources to sustain post-treatment recovery have been sparse, and relapse remains the norm [3].

Many experts have called for routine monitoring as key to detecting symptoms early enough to re-intervene before a lapse into use or relapse into addiction occurs [4,5,6,7,8]. Unfortunately, recovery monitoring is costly to providers, not well-reimbursed, and places burdens on patients. To add to the problem, most measures in common use for SUD monitoring are narrow in focus, and may fail to detect biological, psychological, and social adjustment factors that put recovery at risk.

This paper describes an approach to comprehensive recovery monitoring that serves modern goals of precision medicine and individualized care. We describe barriers to comprehensive monitoring and weaknesses in commonly used measures, and we explain how modern measurement techniques can overcome those problems. We describe the development of two modern measurement systems for monitoring general medical recovery developed by the National Institutes of Health; The Patient-Reported Outcomes Measurement Information System (PROMIS) and the NIH Toolbox. Finally, we explain how those measurement systems can be applied economically and effectively to enhancing SUD recovery success.

## 2. Advances in Chronic Care Medicine

The NIH Human Genome project has created a paradigm shift among healthcare professionals eager to exploit a model of individualized care—precision medicine—which enables individualized treatments tailored to the specific genetic make-up of each patient [9]. In the future, instead of everyone receiving the same medication, diet, surgery, psychotherapy, or recovery support, interventions will take advantage of the unique character of each patient’s biology (genotype), psychosocial history, signs, and symptoms (phenotype) to maximize treatment outcomes. There are already examples of interventions that have restored physical health by altering a patient’s genetic activity to prevent cancer death, e.g., [10].

Together with the renaissance in genetics has been progress in neuroscience enabled by linking modern brain imaging techniques with genetics [11]. Models of how the brain processes, retains, and retrieves information have evolved from biochemical processes involving neurons and synapses to ones that acknowledge constant change in our genetic makeup arising from a complex of biopsychosocial processes e.g., [12,13] that enable those neurons to achieve cognitive-emotional functions. The integration of genomics and neuroscience has greatly advanced our understanding of the recovery process. With regard to alcoholism and other substance use disorders (SUD), there has been little doubt in the past several decades that genes play a role in addiction and recovery [14,15]. When findings in the Human Genome Project began to emerge, it became possible to understand addiction vulnerability in new ways. Understanding the dynamics of genetic influences on the brain began to inform addiction treatment and recovery. Today, it is widely accepted that addiction is a chronic disease. However, we now know that it is not merely a chronic brain disease [16]; it is a genomic disorder that affects the brain, e.g., [17,18].

One of the most challenging aspects of all chronic diseases is that even when treated, recovery can take a long time and require systems of care to sustain long-term health and avoid relapse [19]. This is especially true in the case of SUDs. Current research indicates that attaining sustainable abstinence-oriented recovery continues for three to five years. This is the time required before the odds ratio for relapse plateaus at its lowest level [20,21,22]. If it takes three to five years to achieve addiction recovery, then it is apparent that recovery support monitoring and timely relapse-intervention services are essential tools for helping addicts in recovery to sustain post-treatment SUD abstinence.

This paper describes recent progress in the creation of monitoring tools that align with the goals of individualized medicine. These new tools place a small burden on both clients and providers, which permits routine monitoring on a far broader range of symptoms than current tools allow. Since they are computerized, these modern measures expand options for administration, enabling completion via personal computers, tablet PCs, and smart phones.

## 3. The Need for Recovery Monitoring

Nobody has been more vocal about the need for recovery monitoring than A. Thomas McLellan [1,23]. Along with others [2,24], McLellan has decried the “acute care” model that was, for decades, the norm in SUD treatment. McLellan and others encouraged a shift to a chronic care model that emphasizes regular monitoring that extends for many years after treatment as a means to better detect pending relapse [1,3,25]. In a 2002 editorial, McLellan [23] proposed that monitoring symptoms should begin while still in treatment, and then extend into recovery on a regular basis. Such a model integrates regular recovery status checkups as an essential tool for achieving and sustaining recovery. In addition, monitoring during recovery provides a means of “early detection of potential problems to enable referral to appropriate care and accepting referral back to continue the monitoring and support.” Rudolph Moos [26] observed that frequent monitoring also has the potential to encourage continued engagement in recovery support activities—a point validated in a 2012 review by White of 415 papers addressing recovery [27]. McLellan’s call for recovery monitoring under a chronic care model has been widely accepted as a key adjunct to achieving self-sustainable recovery during the five years following treatment [28]. However, from a measurement perspective, the question arises as to what domains should recovery monitoring measure, how often should recovery status be measured, and by what means should it be measured? According to the World Health Organization (WHO) [29], recovery monitoring should address physical, mental, and social health domains. Frequency of monitoring depends on the specific disease. The means of measurement should include both physical indicators and patient reported outcomes (PROs). Unfortunately, most measurement tools used to monitor SUD recovery inadequately address WHO domains, and due to staff and patient burden tend to be infrequent, if conducted at all.

## 4. Background on Recovery Monitoring in SUDs

Until the turn of the 21st century, addiction treatment and recovery has been guided by various models ranging from criminality, moral weakness, social maladaptation, and mental illness. By the mid 1980s a psychosocial model dominated the addiction field. It assumed that recovery was enabled by addressing addict mental health, and helping those in recovery to learn new behaviors that would (a) avoid social and environmental contexts associated with their life while addicted, and (b) develop appropriate social role engagement, e.g., [30,31,32,33]. This psychosocial model of addiction and recovery was based primarily on behavioral science and clinical trial-and-error over many decades [34]. Although the psychosocial model makes sense, therapeutic outcomes were disappointing, and relapse remained the norm for the vast majority of addicts who sought treatment [3].

As early as 1977, George Engel [35] urged psychiatry to shift from a medical model to a biopsychosocial model. Smith and Nicossio [36] similarly urged psychology to make the same shift. However, with regard to addiction, the bio- part of the model has focused mostly on the physical symptoms of withdrawal and craving.

The role played by biology in addiction and recovery began to expand when George Koob and Michele Le Moal [37] introduced a biopsychosocial model that incorporated emerging knowledge in genetics and neuroscience. The Koob and Le Moal model integrates modern genomics and neuroscience to help the addiction field better understand the physiological dynamics that sustain craving and maladaptive behaviors for years following treatment and how biological factors influence psychological and social behaviors. Koob and LeMoal argued that both the development of SUD, and the process of recovery from it, involve re-setting the homeostatic set-point of an addict’s gene expression caused by the neurotoxicity of alcohol and drugs. Biologists refer to the re-setting process as “allostasis,” *i.e.*, the process of re-achieving self-regulating homeostatic stability of bodily functions through durable physiological and behavioral change.

## 5. Allostasis and Recovery

There are basically two models of recovery. Abstinence-oriented recovery (AOR) is the most common type of recovery. AOR involves complete abstinence from addictive substances. Thus, patients will experience a range of symptoms beginning with withdrawal as allostasis progresses over the period of five years or so that self-sustainable abstinence can take to achieve. A second model of recovery is medically-maintained recovery (MMR), which relies upon medication, such as opiate replacement therapy (ORT) [38] that enables the body to avoid withdrawal symptoms and craving. The same factors shown to trigger relapse among those in AOR can trigger relapse among those in MMR. Patients who abuse drugs while in MMR will disrupt the homeostasis maintained by their medication. Likewise, MMR patients whose symptoms indicate the need to adjust the dose of their medication are at risk of experiencing allostasis symptoms and withdraw from treatment if medication is not adjusted in time.

Regardless of recovery model, addiction recovery spans the period of abstinence allostasis needed to restore homeostasis under AOR or the period under MMR that avoids allostasis through medication—possibly for the remainder of one’s life. In both cases, regular monitoring of diverse symptoms offers a way to detect and intervene when relapse risk has increased.

Diverse symptom measurement is important because the complexity of an addict’s brain and genome ensures that addiction and recovery will always be idiosyncratic [17,18]; see [39]. No two addicts will experience the same allostasis. The nature of symptoms that might indicate a potential lapse (brief return to substance use) or that might lead to relapse (a return of addictive use) will be a function of intersecting emotional, social, and biological changes. Thus, vulnerability to relapse cannot be determined without comprehensive, routine assessments.

## 6. The Challenges of Recovery Monitoring

### 6.1. Provider Burdens

The proverbial 900-pound gorilla that impedes frequent recovery monitoring is the measurement burden for both providers and patients [31,32]. Monitoring places a substantial financial burden on addiction health service providers, in part, because most measures in wide use require costly training to ensure validity and appropriate interpretation of results, e.g., [33]. Additionally, clinical staff must take time away from service tasks to administer, score, interpret, and feedback information to patients in recovery. Until recently, monitoring was rarely reimbursed [40]. Even under new laws requiring third-party coverage of substance use disorders and mental illness, reimbursement for recovery monitoring is not fully compensated [41]. As a consequence, when monitoring is done, it is not done often enough, and with the exception of funded research experiments, measures used are typically narrow in scope and, therefore, limited in their ability to detect change that might trigger relapse in time to intervene successfully.

### 6.2. Patient Burdens

While staff burden often creates barriers to recovery monitoring, the most frequently discussed burden is the one monitoring places on patients [42]. Even brief recovery monitoring measures normally require that patients schedule a visit to a specific location at a specified time to complete questionnaire-guided interviews or to fill out questionnaires, and then wait to have them scored and interpreted. Brief questionnaires or interviews tend to be narrow in scope. They might strike recovering patients as superficial and a waste of time, thereby lowering participation and raising vulnerability to undetected lapses and eventual relapse.

### 6.3. Limited Access to Monitoring

Frequent, periodic, comprehensive monitoring is not a national norm [3,41]. Even when recovery support services are available, a great many patients fail to participate [8,43], or they enroll but drop out in less than a year [3,44,45,46,47,48]. Unfortunately, research shows that it takes at least a year to realize any benefit from participation in recovery support [3,43].

## 7. Recovery Monitoring Measures

### 7.1. Measurement Problems

The time-consuming length of most recovery monitoring measures is a legacy of the acute-care model of treatment that dominated the field for many decades. Measures for monitoring SUD recovery have been for the most part based on abbreviations of lengthy diagnostic assessments administered during treatment intake. Thus, even if a chronic-care model has been adopted, the measures used to implement it likely are derivations from older, acute-care legacy measures in wide use for diagnosis and treatment planning.

Here is a summary of the problems associated with the short-form recovery monitoring measurement approach:

(1) The goal of diagnosis for treatment is not necessarily the goal of recovery monitoring. As such, most measures are skewed toward high severity of symptoms. This characteristic makes them less sensitive for detecting levels of wellness.
(2) Shorter forms reduce measurement sensitivity and precision, in part because of the reduced range of symptoms addressed. To broaden the scope of measurement, it is necessary to employ additional short forms, which will increase patient burden and provider costs.
(3) The validity of short form measures is dependent on a response to every item. It is not possible to skip seemingly irrelevant or tedious items. This might affect the face validity needed to sustain patient engagement over many years.
(4) Scoring and interpretation of abbreviated diagnostic measures still requires some degree of formal training, and will also consume staff time to score and interpret.
(5) Administration costs can reduce the frequency of monitoring.
(6) Patient burden is increased by use of paper-and-pencil measures or questionnaire-guided interviews because they normally must be completed at a location and time that can interfere with patient role responsibilities such as training or work. That burden risks skipped sessions and incomplete trend data, undermining the point of monitoring to detect lapse situations.
(7) Most paper-and-pencil monitoring measures require the creation of trend graphs by hand if any are to be used. This additional burden may hamper the use of trend information.
(8) Most recovery monitoring measures do not adequately address biological symptoms likely to appear during allostasis.

### 7.2. Measurement Solutions

In 2003, the National Institutes of Health, in consultation with health outcomes experts, initiated a national program of research to address the shortcomings of recovery monitoring measures in general healthcare. That system became the basis for development of modern measures that became the NIH Patient-Reported Outcomes Management Information System (PROMIS). PROMIS was intended to take advantage of modern measurement theories and ready access to computer tools to develop a comprehensive battery of recovery monitoring measures that could overcome the shortcomings listed above. Below is a list of recovery monitoring characteristics researchers believed would overcome the many shortcomings of existing measures [49,50,51,52].

(1) Measures should be obtainable at regular intervals to establish baseline status and enable meaningful indicators of change.
(2) Measures should be brief enough to not be viewed as burdensome to the patient, address a broad enough spectrum of symptoms to seem relevant to the patient’s experience with recovery, and therefore sustain engagement over the many years of recovery.
(3) Precision and sensitivity should not be sacrificed for the sake of brevity.
(4) Ideally, measures should enable patients in recovery to complete them on their own, at a place and time of their convenience without the need for administration by a trained service provider.
(5) Measures should not require a significant training burden to recovery support staff to administer and interpret results, as training costs can exclude staff not yet trained, and weaken outreach and support decisions when intervention is indicted by scores.
(6) Measures should be self-scoring in the sense that results would be instantly recorded and converted to easily-understood trend results for each variable monitored.
(7) Measures should be broad enough to be sensitive to changes in general physical and mental health as well as social functioning.
(8) Measures should be modifiable or expandable without a significant increase in burden to address the individualized needs of patients in recovery such as addressing exacerbating conditions like physical health or disability, significant chronic psychiatric conditions, or housing needs.
(9) Measures should be valid for all patients regardless of race, gender, age, or disease.
(10) The cost of monitoring should be limited to initial integration with existing record systems, and staff time to examine and interpret trends for sustained improvement or lapse.

## 8. The Promise of PROMIS for Recovery Monitoring

An overarching goal for PROMIS was to develop patient-reported outcome (PRO) measures that would be valid regardless of patient characteristics such as age, sex, education, or ethnicity, as well as disease or disorder. For example, pain, fatigue, and social role disruption are common to most diseases and disorders. Validating items relevant to a broad spectrum of chronic illness enables PROMIS to achieve several important research and clinical goals. PROMIS can help to reduce measurement error in comparative research that examines symptoms such as pain which is present across a variety of conditions [53]. PROMIS also adds clarity to communication among health service providers when discussing symptoms common to many patient types, diseases, and disorders. Finally, PROMIS offers measures designed to advance SUD recovery [54].

### 8.1. NIH PROMIS

**Figure 1:**
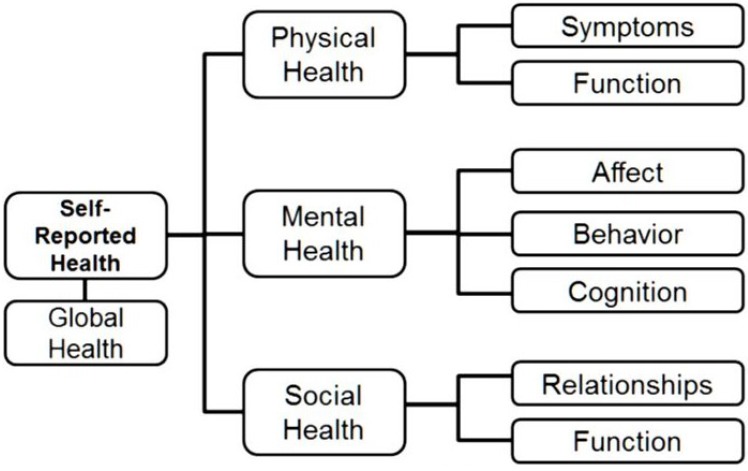
PROMIS Domain/Topic Framework.

By 2010, NIH PROMIS had delivered on its promise to the field of healthcare by delivering a set of computerized patient-reported outcome (PRO) general health measures based on modern measurement theory (MMT) to monitor changes in biological, psychological, and social health status quickly and precisely. The original domain framework, presented in Figure 1, followed recommendations by experts and the World Health Organization [55]. During its initial phase, PROMIS researchers developed item banks for 12 general health domains/topics listed in Table 1. Not all items in a bank are presented to patients because MMT measures use computerized adaptive testing (CAT) for administration (see below).

NIH PROMIS offers recovery-monitoring measures that meet the individualization needs of modern precision medicine models. They are brief (often two minutes or less per domain/topic), available royalty-free, measure the same domains/topics that lengthy intake assessment interviews and questionnaires target, and they exhibit validities equal to or better than older legacy measures. Moreover, they are available in a variety of languages for patients not fluent in English.

**Table 1:** PROMIS Core Health Domains/Topics.

Domains	Items in Bank
Emotional Distress—Anger	29
Emotional Distress—Anxiety	29
Emotional Distress—Depression	28
Fatigue	95
Pain—Behavior	39
Pain—Impact	41
Physical Function	125
Satisfaction with Discretionary Social Activities	12
Satisfaction with Social Roles	14
Sleep Disturbance	27
Sleep-Related Daytime Impairment	16
Global Health	10

### 8.2. PROMIS Assessment Center

The primary burden on addiction health service providers to incorporate PROMIS measures is to take the time to register and establish an account at the PROMIS Assessment Center at Northwestern University [56]. Once registered, providers can integrate results with their record system, enroll patients and staff, select the domains/topics desired for each patient, and arrange for secure Internet access (normally via a home or clinic PC or tablet). Computerization means that for most patients in recovery, scheduling time, and travel for monitoring is minimized or periodically eliminated. Computerization enables fast data collection and prompt scoring that can provide feedback that includes trend data for each domain/topic measure on a nationally-normed metric that ranges from severe symptomatology to a high degree of functioning and wellness. Recovery support service providers can, as circumstances warrant, debrief patients in recovery via free video conference systems such as Skype or Viber.

### 8.3. NIH Toolbox

For most needs in addiction health services, PROMIS offers a sufficient variety of measures to enable an individualized-medicine approach to both treatment and recovery support of most diseases. When necessary, PROMIS measures can be augmented by banks from the NIH Toolbox to address cognitive and emotional conditions. NIH Toolbox measures were developed in response to the 2006 *NIH Reform Act*. Toolbox measures are also accessible through the PROMIS Assessment Center and NIH Toolbox [57]. Table 2 summarizes Toolbox CAT measures available.

**Table 2:** * NIH Toolbox Domains/Topics.

Domains	Items in Bank
Negative Affect-Anger	22
Negative Affect-Fear	29
Negative Affect-Sadness	27
Well-Being—Life Satisfaction	10
Well-Being—Meaning & Purpose	18
Well-Being—Positive Affect	34
Stress	10
Stress & Self-Efficacy	10

* Adapted from NIHToolbox.org.

## 9. The Enabling Technology behind PROMIS and Toolbox

### 9.1. Classical Test Theory

Most measures requiring paper-and-pencil forms or questionnaire-guided interviews were developed using Classical Test Theory (CTT) [58]. These measures contain a sample of items believed by experts to reflect the range of symptoms for a target domain/topic. CTT measures are validated by examining the relationship between the aggregate item scores and independently observed symptoms or other indications of disease or disorder. A basic assumption of CTT is that positive and negative measurement errors are averaged out if the pool of items is large enough [59]. As a result, the number of items can be quite large. Scores dependence on aggregated item responses, meaning that skipped items reduce precision and validity. As mentioned earlier in this paper, when instrument developers try to reduce the burden of a CTT measure it is usually by eliminating items. That decision can affect measurement precision and it requires revalidation of the abbreviated questionnaire.

### 9.2. Modern Measurement Theory

Modern psychometric methods following MMT rely on item-level validation using Item Response Theory (IRT). IRT was first introduced in the 1950s by Frederick Lord [60] and Warren Torgerson [61], and its impact became greater with improvements in computerized adaptive technology. In 1976, the first large-scale CAT application based on IRT was introduced—the Armed Services Vocational Aptitude Battery (CAT-ASVAB) [62]. CAT-ASVAB dramatically reduced military recruit testing time while improving classification precision. It produced a huge reduction in costs to administer and score. Unfortunately, wider applications of CAT technology were stymied by limited Internet access, computational capacity, and the sheer size of computers themselves. It was not until the turn of the 21st century that technology had evolved to the point that CAT has become a practical alternative to burdensome CTT measures.

### 9.3. How CAT Works

CAT reduces the number of items presented without sacrificing validity and precision. Respondents are presented independently-validated items from a pool of possible items (item banks) based on previous question responses. Each item in the pool is calibrated on severity level, from highly symptomatic to a high level of well-being. Similar to a hearing test, the computer first presents an item of average endorsement difficulty, such as “I drank too much” (with response options on a 1–5 frequency scale). If the response is “Often,” the algorithm selects an item of greater difficulty such as “It was difficult for me to stop drinking after one or two drinks.” Once a high-severity item can no longer be endorsed, the program moves to the next domain/topic similar to a hearing test moving to the next frequency. Conversely, if the first response cannot be endorsed, the computer chooses an item easier to endorse (*i.e.*, less symptomatic) and proceeds in the opposite direction. In cases where responses are ambiguous, additional items of similar difficulty are presented in order to achieve precision. Most PROMIS banks require 4–6 items and take under two minutes to obtain a precise score using CAT.

## 10. The Application of PROMIS to Addiction Treatment and Recovery

### 10.1. Patient Engagement

Patient engagement is key to successful recovery support [63]. As mentioned earlier, many people suffering from SUD have few if any recovery-support options, most fail to enroll in aftercare when available, or enroll but drop out before they can get any benefit from aftercare. Done right, monitoring offers one way to help keep patients engaged in recovery support services, but it must be timely, perceived as valuable by patients, and lead to prompt and appropriate re-interventions [4,5,64]. Since allostasis progresses in a diverse and often unpredictable fashion, the symptoms experienced during recovery may not fit the narrow scope of most currently used measures, and thus can appear to contribute no meaningful information to help sustain recovery. Thus, engagement can be enhanced when monitoring items ask more than the current focus on craving, criminality, and mental health [54].

### 10.2. Diverse Content

As Scott, Dennis, and colleagues have shown [65,66,67], the paths to both addiction and recovery are diverse, idiosyncratic, and dynamic. During the long duration of allostasis in recovery, many life changes can trigger relapse if not detected and addressed. For example, many people recovering from SUD experience pain, both physical and emotional. For many, pain was the major reason they began drinking and/or taking addictive drugs. In addition, the *sturm und drang* of daily life is accompanied by frustrations and unpleasant events that can increase anger, anxiety, or depression. Emotional reactions to daily experiences can also affect sleep quality that can spill over into wake disturbances manifested in drowsiness, irritation, fatigue, and inattention. These symptoms, if allowed to cascade, can interact to trigger relapse; but they might go unnoticed using conventional monitoring measures.

### 10.3. Self-Management

Self-management has become a key element in modern recovery medicine [28]. Moreover, self-administration reduces the measurement burden for providers, and provides greater flexibility in time and location for patients to participate. The ability of PROMIS to generate trend graphs on a common metric also offers easily understandable information about change over time. Trend data can help providers develop more precise priorities for recovery interventions and activities, while offering clear evidence of progress or the need for re-intervention to patients [5]. Finally, following general medical principles for managing chronic illness [68], PROMIS monitoring helps place the patient at the center of their course of recovery, in part, by strengthening the temporal link between their PRO trend data and change.

### 10.4. SUD-Specific Measures

Finally, combining core PROMIS general health measures with PROMIS alcohol and substance use measures broadens the scope of recovery monitoring and takes up the same amount of time or less than narrower, and less-precise brief paper-and-pencil or questionnaire-guided interview measures. PROMIS can provide useful insights into the recovery status of an individual patient, and provides information useful in choosing an appropriate and timely response to ward off SUD relapse.

## 11. PROMIS Alcohol and SUD Measures

During the second phase of the PROMIS project, NIH funded the development of item banks addressing symptoms specific to alcohol and drug abuse. In the development of the alcohol and substance use banks, researchers systematically assembled more than 6400 legacy items used in SUD treatment and research [69]. Teams of experts eliminated redundant, ambiguous, or confusing items, sorted them into sub-domain topics, and then asked clinicians and patients if they overlooked anything. The resulting smaller pool of items was then administered to people being treated for alcoholism and other SUDs as well as drinkers and drug users from the community who were not seeking treatment. IRT analyses eliminated items that functioned differently for subgroups of respondents, and identified where on the severity metric an item fell. Due to the fact that substance use impairs cognitive functioning, wording was kept simple. The Flesch-Kincaid readability [70] for all alcohol and SUD banks was no higher than third grade.

More detailed information regarding methodology is available in Pilkonis, *et al*. [71,72]. Table 3 lists the domains/topics for the alcohol and SUD banks, together with the number of items. In the case of both alcohol and SUD, the substance use banks are likely to be of most interest to researchers and clinicians as these banks incorporate frequency of use and severity of craving. Additional sub-domain banks would be most useful for addressing research questions, but they also might be germane to recovery management when issues arise in therapeutic interactions.

**Table 3:** * PROMIS Alcohol and Substance Use Disorder Domains/Topics.

Domains	Items in Bank
Alcohol Use	
Alcohol Use	37
Negative Consequences	31
Positive Consequences	20
Negative Expectancies	11
Positive Expectancies	9
Substance Use Disorder	
Severity of Use	37
Positive Appeal of Use	18

Notes: * Adapted from Pilkonis, *et al*. [50] and Pilkonis, *et al*. [51].

Items for alcohol use and consequences were written in a first-person, past-tense format with a 30-day time frame and five response options reflecting frequency. Items for expectancies were written in a third-person, present-tense format with no time frame specified and five response options reflecting intensity. Initial calibrations of the SUD items were similar for 30-day and three-month time frames. Final calibrations used data combined across the time frames, making the items applicable with either interval. One might expect that during treatment and the first year of recovery, a 30-day response frame would be preferred by health service providers; whereas the longer timeframe might be more appropriate once a longer pattern of abstinence/maintenance has been achieved.

## 12. The Future of PROMIS Recovery Monitoring

Since CAT measures are computerized, they can be integrated with existing electronic health record systems. This offers providers some economy of scale, and it enables other benefits such as producing graphical summaries, integrating results with other reports and records, and providing near instantaneous feedback. Many clinical settings already administer PROMIS using tablet PCs or desktop PC carrels while patients sit in the waiting room for appointments. Having immediate access to PROMIS scores and trends facilitates checkups by helping providers focus on symptom changes since the previous encounter, and it helps to ensure that symptoms are not overlooked. Patients often do not volunteer symptom information simply because the doctor did not ask [73].

An exciting potential of CAT measures is integration with smartphones. Accessing PROMIS measures for recovery monitoring via smartphone is not breaking new ground. There are already several SUD applications under study that perform ecological momentary assessment (EMA) [74] to detect craving, and connect recovering patients to appropriate intervention (ecological momentary intervention; EMI) [75,76]. Recently, Chestnut Health Systems has begun studying a smartphone EMI application for substance abusing adolescents [77].

However, EMA has recently come under criticism as too narrowly focused and sometimes overly burdensome to patients, see [78]. In addition, EMA smartphone systems only address a few topics (like craving), and positive effects on health outcomes dissipate upon cessation [79]. Anecdotally, some patients have associated their smartphones with therapeutic benefits for sustaining recovery. They indicated that games on smartphones provided distraction. As one patient noted, absorption in the game leads to forgetting about cravings [80]. This phenomenon was recently validated in a 2015 study by Skorkas-Brown [81]. Thus, many patients in recovery are already using their smartphones as aids to sustained recovery even without EMA, EMI, or PROMIS.

It remains easier to complete computerized questions on an appliance with good screen readability such as a desktop or tablet PC. Clearly, integration of PROMIS with other smartphone applications such as EMA/EMI should be examined with care so as not to undermine measurement validity. The PROMIS Assessment Center is releasing an application for iPhones through iTunes in 2015 [82]. Hopefully that work will expand to Android smartphones and tablet PCs in the near future.

Now that PROMIS general health measures can be augmented with SUD-specific domain banks, the system offers the ideal recovery monitoring tool. Relatively-speaking, PROMIS will normally be faster, better, and cheaper to use than widely-used paper-and-pencil or questionnaire-guided interviews currently in use. It represents the state-of-the-art in terms of speed, precision, and sensitivity with no demonstrated loss in validity compares to short forms and their longer legacy measures used in assessment for diagnostic and treatment planning purposes.

## 13. Discussion

Our goal has been to inform readers about precise, low-burden measures for assessing and monitoring recovery from alcoholism and other SUD that are royalty-free. These new measures can be found in the NIH PROMIS and NIH Toolbox systems maintained at the Assessment Center at Northwestern University. A unifying rationale is that application of individualized medicine models, even without gene-based interventions, is possible with these measures. Frequent progress monitoring using measures broad enough in scope to capture signals of relapse creates opportunities to re-intervene. Use of these tools in support of individualized medicine meets the monitoring goals that have long been called for in the literature but that have not been widely implemented due to cost and other considerations.

Some researchers and healthcare providers may have delayed implementing PROMIS because they were unaware that CAT PRO measures addressing symptoms of alcohol and SUD abuse are available. In fact, the SUD banks are just now coming on line. For patients with multiple morbidities, NIH Toolbox offers complementary CAT item banks that address a variety of cognitive and emotional domains/topics.

Integrating CAT measures into existing assessment systems makes sense because they are scored automatically and require no formal training to administer. Even though interpretation of assessments will still require trained professionals, the time devoted to information collection can be substantially reduced using CAT measures. Given the reduced patient burden of CAT measures coupled with expanded scope, PROMIS and Toolbox offer ample reasons to initiate frequent recovery monitoring to enable individualized medicine services now. Continued use of paper-and-pencil measures offers comparatively less precision to detect relapse while potentially disengaging patients from aftercare participation. In summary, there is adequate justification to adopt or switch to NIH CAT measures to support SUD recovery.
